# Hierarchical scaffolding of an ERK1/2 activation pathway

**DOI:** 10.1186/1478-811X-11-65

**Published:** 2013-08-29

**Authors:** Susanne Vetterkind, Ransom H Poythress, Qian Qian Lin, Kathleen G Morgan

**Affiliations:** 1 Department of Health Sciences, Boston University, Boston, MA 02215, USA; 2 Department of Health Sciences, Boston University, 635 Commonwealth Ave, Boston, MA 02215, USA

**Keywords:** Actin, Extracellular signal regulated kinase, Mitogen activated kinase, Scaffold protein, Phorbol ester

## Abstract

**Background:**

Scaffold proteins modulate cellular signaling by facilitating assembly of specific signaling pathways. However, there is at present little information if and how scaffold proteins functionally interact with each other.

**Results:**

Here, we show that two scaffold proteins, caveolin-1 and IQGAP1, are required for phosphorylation of the actin associated pool of extracellular signal regulated kinase 1 and 2 (ERK1/2) in response to protein kinase C activation. We show by immunofluorescence and proximity ligation assays, that IQGAP1 tethers ERK1/2 to actin filaments. Moreover, siRNA experiments demonstrate that IQGAP1 is required for activation of actin-bound ERK1/2. Caveolin-1 is also necessary for phosphorylation of actin-bound ERK1/2 in response to protein kinase C, but is dispensible for ERK1/2 association with actin. Simultaneous knock down of caveolin-1 and IQGAP1 decreases total phorbol ester-induced ERK1/2 phosphorylation to the same degree as single knock down of either caveolin-1 or IQGAP1, indicating that caveolin-1 and IQGAP1 operate in the same ERK activation pathway. We further show that caveolin-1 knock down, but not IQGAP1 knock down, reduces C-Raf phosphorylation in response to phorbol ester stimulation.

**Conclusions:**

Based on our data, we suggest that caveolin-1 and IQGAP1 assemble distinct signaling modules, which are then linked in a hierarchical arrangement to generate a functional ERK1/2 activation pathway.

## Background

Scaffold proteins facilitate the assembly of signaling cascades by simultaneous binding of several consecutive components of a signaling pathway. By doing so, they regulate speed, specificity, intracellular localization and amplification of signal propagation (for review, see [1]). Scaffold proteins for the mitogen activated protein kinase (MAPK) cascade were among the first to be discovered [2,3]. The expanding group of MAPK scaffolds includes many scaffolds for the extracellular signal regulated kinase (ERK) pathway, such as kinase suppressor of Ras1 (KSR1), paxillin, MEK partner 1 (MP1), IQ motif containing GTPase activating protein 1 (IQGAP1) and caveolin-1 [4,5].

The canonical ERK pathway consists of three kinase tiers: Raf, MEK (MAPK and ERK kinase) and ERK. ERK/MAPK scaffolds, in the narrow sense, assemble two or all three tiers of the canonical ERK pathway (Raf, MEK and ERK), thus - when expressed at optimal stoichiometry - facilitating and accelerating ERK activation, but at the same time restricting signal amplification. KSR1, which binds to Raf, MEK and ERK, belongs to this category. Scaffold proteins in the broader sense associate with one or more members of the MAPK pathway within a larger complex or protein platform, such as paxillin, which interacts with Raf, MEK and ERK within the focal adhesion complex [6]. Another example of this category is caveolin-1, the characteristic membrane protein of caveolae, which associates with many signaling proteins including protein kinase C (PKC), Ras, Raf MEK and ERK at the caveolar membrane [7-11].

Although much is known about interaction, function and regulation of the various scaffolds, there is at present little information if and how MAPK scaffold proteins functionally interact with each other. Since most studies focus on only one scaffold protein, the available literature concerning scaffold proteins appears to give the impression that most scaffolds function autarkically, i.e. independently of other scaffolds.

In smooth muscle, ERK1/2 activation can lead to different signaling outcomes ranging from proliferation to contraction, depending on the stimulus. In an effort to unravel stimulus-specific ERK1/2 signaling, we have recently shown that ERK1/2 is divided into subfractions in aortic smooth muscle cells, and that these subfractions respond differently to distinct signaling cues [11]. In particular, we found that an actin associated fraction of ERK1/2 is phosphorylated and remains bound to actin after PKC stimulation, whereas serum stimulation leads to reduced actin association of ERK1/2. Caveolin-1, a known regulator of ERK1/2 activity [12,13], was found to be critical for stimulus-specific phosphorylation of actin-associated ERK1/2, however, the mechanism for this association was not clear.

Here, we hypothesized that in addition to caveolin-1, a second scaffold protein is necessary to maintain ERK1/2-actin association during PKC stimulation. In the present study, we identify the actin-binding IQGAP1 as the ERK1/2 scaffold that targets ERK1/2 to the actin cytoskeleton. Our data show that for phosphorylation of actin-associated ERK1/2 in response to PKC activation, both caveolin-1 and IQGAP1, in a serial arrangement, are required. Thus, our results demonstrate that the hierarchical nature of scaffolding is an important concept to consider in understanding signaling pathways.

## Results and Discussion

### Stimulus-specific localization of activated ERK1/2 is not based on increased actin binding of ERK1/2

**Figure 1:**
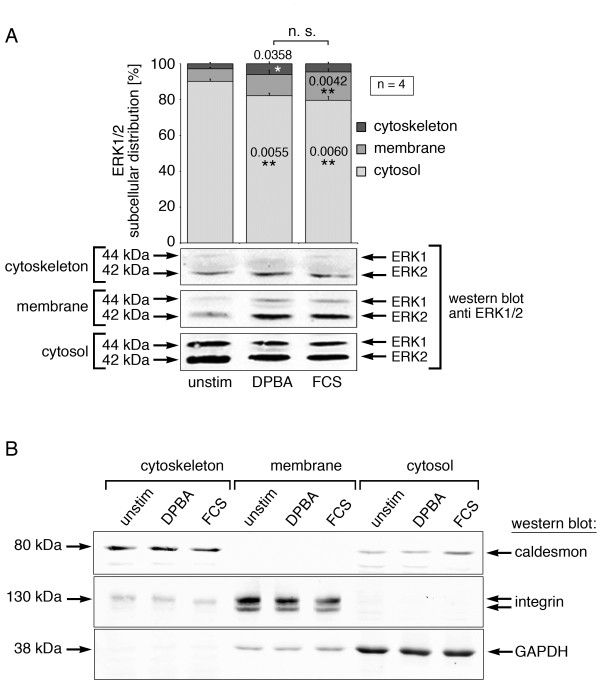
**ERK1/2 subcellular localization does not show stimulus-specific differences.** A7r5 cells were stimulated for 5 minutes with either a phorbol ester (DPBA) or with serum (FCS), or left unstimulated before subcellular fractionation by differential ultracentrifugation. Samples were analyzed by western blotting. **(A)** Average ERK1/2 subcellular distribution from four independent experiments is shown along with a typical western blot. **(B)** Control staining with marker proteins for the cytoskeletal fraction (caldesmon), the membrane fraction (integrin) and the cytosolic fraction (GAPDH). Please note that the integrin band appears as a doublet. Significance (*compared to unstimulated samples) and p-values are indicated on chart. n.s., not significant; error bars represent standard errors.

We have recently shown that an actin-associated fraction of ERK1/2 is activated in a stimulus-specific manner in vascular smooth muscle cells [11]. Moreover, ERK1/2 has been shown by our group to bind to actin directly [14]. To determine whether ERK1/2 binding to actin is modulated differentially by stimulation, we subjected unstimulated or stimulated A7r5 lysates to subcellular fractionation by differential ultracentrifugation. Cells were stimulated with either fetal calf serum (FCS), a proliferative stimulus that leads to solubilization of cytoskeletal ERK1/2 [11], or the phorbol ester 12-deoxyphorbol 13-isobutylate 20-acetate (DPBA), which causes cytoskeletal rearrangements and podosome formation [15] as well as phosphorylation of cytoskeletal ERK1/2 [11] via activation of PKC. We chose a treatment duration of 5 minutes for all experiments, since in our previous work, the earliest stimulus-specific effects appeared at this time point [11]. As shown in Figure 1A, ERK1/2 is found mainly in the cytosolic fraction after differential ultracentrifugation, indicating that either actin binding affinity is low, or only a small ERK1/2 fraction interacts with actin directly. We detected a small but significant redistribution of cytosolic ERK1/2 into the membrane and cytoskeletal fractions upon serum or phorbol ester stimulation, but no significant differences between the two types of stimulation. Marker protein staining shows proper segregation of the cytoskeletal, membrane and cytosolic fractions (Figure 1B). These results indicate that stimulus-specific localization of phosphorylated ERK1/2 to actin filaments is not mediated by increased binding of ERK1/2 to actin filaments. We conclude that, in this case, ERK1/2 is predistributed in distinct locations in the cell, positioned to be activated by distinct stimuli, rather than being targeted from a common pool to a specific subcellular localization upon stimulation.

### ERK1/2 interactions with its scaffolds are stimulus-specific

**Figure 2:**
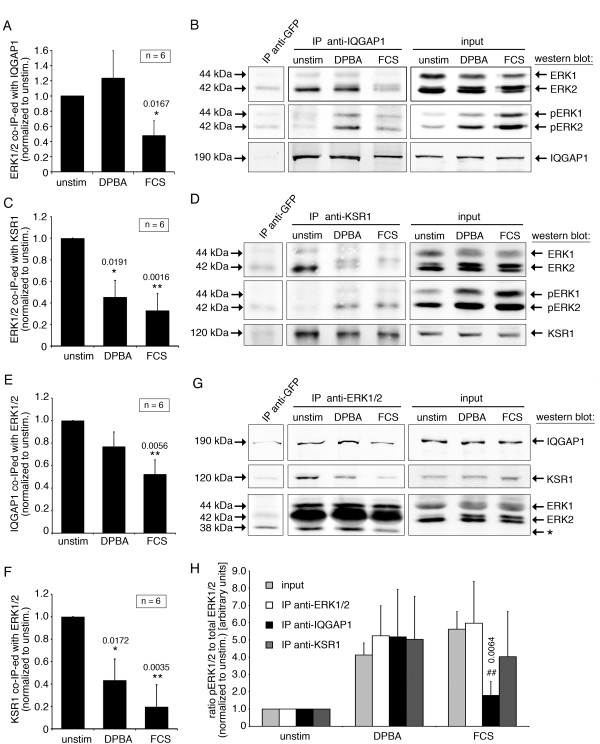
**IQGAP1 and KSR1 interact with ERK1/2 in a stimulus-specific manner. (A)** Lysates of unstimulated, DPBA-stimulated or FCS-stimulated cells were subjected to immunoprecipitation (IP) experiments. Co-immunoprecipitated proteins were detected by western blotting and analyzed by densitometry. **(A)** The graph shows average band intensities of ERK1/2 co-immunoprecipitated with an anti-IQGAP1 antibody. **(B)** Representative western blot for an anti-IQGAP1 IP experiment. **(C)** Average band intensities of ERK1/2 co-immunoprecipitated with an anti-KSR1 antibody. **(D)** Representative western blot for an anti-KSR1 IP experiment. **(E)** Average band intensities of IQGAP1 co-immunoprecipitated with an anti-ERK1/2 antibody. **(F)** Average band intensities of KSR1 co-immunoprecipitated with an anti-ERK1/2 antibody. **(G)** Representative western blot for an anti-ERK1/2 IP experiment. Please note that a non-specific band of 38 kDa (*) was seen in ERK1/2 IPs. **(H)** Analysis of pERK to total ERK ratios in input samples as well as in immunoprecipitates. Significance (*compared to unstimulated samples; # compared to ) and p-values are indicated on chart; error bars represent standard errors. IP, immunoprecipitation. Please note that ERK2 appears as a doublet after stimulation, with the upper band representing phosphorylated ERK2.

We next hypothesized that stimulus-specific activation of ERK1/2 is mediated by scaffold proteins. To identify possible changes in ERK1/2 interaction with its scaffolds during stimulation, we carried out endogenous ERK1/2 co-immunoprecipitation experiments with unstimulated, DPBA- or FCS-stimulated cells, and looked for changes in co-precipitation of several known ERK1/2 scaffolds including paxillin, KSR1, IQGAP1 and basic calponin. In the cases of IQGAP1 and KSR1, we found changes in ERK1/2 interaction upon stimulation, and added co-immunoprecipitation experiments in the reverse direction (anti-IQGAP1 and anti-KSR) as control (Figure 2). Representative western blots are shown in Figure 2B,D and F. Statistical analysis of densitometry data from five to six independent experiments for each IP revealed that: (1) binding between ERK1/2 and IQGAP1 is significantly reduced only after serum, but not after phorbol ester stimulation (Figure 2A,E) and (2) binding between ERK1/2 and KSR1 is significantly reduced after both, phorbol ester and serum stimulation (Figure 2C,G). These findings show that ERK1/2 interaction with its scaffolds KSR1 and IQGAP1 is modulated by stimulation of the cells, resulting in a stimulus-specific interaction profile.

To find out whether in addition to ERK1/2 binding, also the ability of either KSR1 or IQGAP1 to activate ERK1/2 changes in a stimulus specific manner, we have analyzed the phospho-ERK1/2 to total ERK1/2 ratios (pERK:tERK) in the immunoprecipitates. In IQGAP1 immunoprecipitates, the pERK:tERK ratio after phorbol ester stimulation reflects the pERK:tERK ratio found in input samples (Figure 2H), which is in agreement with IQGAP1 supporting ERK1/2 phosphorylation after phorbol ester stimulation. After serum stimulation however, the pERK:tERK ratio is significantly lower compared to the input samples. In ERK1/2 immunoprecipitates as well as in KSR1 immunoprecipitates, the pERK:tERK ratio is not significantly different from the pERK:tERK ratio in the input samples.

The different abilities of IQGAP1 and KSR1 to bind phospho-ERK1/2 after serum stimulation could reflect different mechanisms of regulation between IQGAP1 and KSR1 regarding their scaffolding activity. KSR1 is subject to a negative feedback regulation by binding of activated ERK1/2 via DEF motifs [16], which reduces KSR1 scaffold activity by impairing interaction between KSR1 and B-Raf, and by displacing KSR1 from the cytoplasma membrane. This feedback mechanism could explain residual binding of phospho-ERK1/2 to KSR1 under conditons that reduce interaction between KSR1 and ERK1/2. It is not known whether a similar feedback meachnism based on phosphorylated ERK1/2 exists for IQGAP1. However, published data support regulation of IQGAP by confromational changes induced by ligand binding [17-20]. Interestingly, IQGAP1 has recently been reported to interact with the chaperones melusin, which is expressed in heart and skeletal muscle, and HSP90 [21]. Though melusin is not expressed in smooth muscle [22], other chaperones might influence IQGAP1 interactions in a stimulus-depending manner to regulate its ligand binding. It is therefore possible that in our cell model, after serum stimulation, IQGAP1 scaffolding activity towards the Raf/MEK/ERK1/2 pathway is reduced by conformational changes of IQGAP1 that inhibit binding of ERK1/2 or phospho-ERK1/2, rather than inhibitory feedback regulation induced by phospho-ERK1/2 binding.

### Knock down of ERK1/2 scaffolds reveals stimulus-specific ERK1/2 activation by IQGAP1

**Figure 3:**
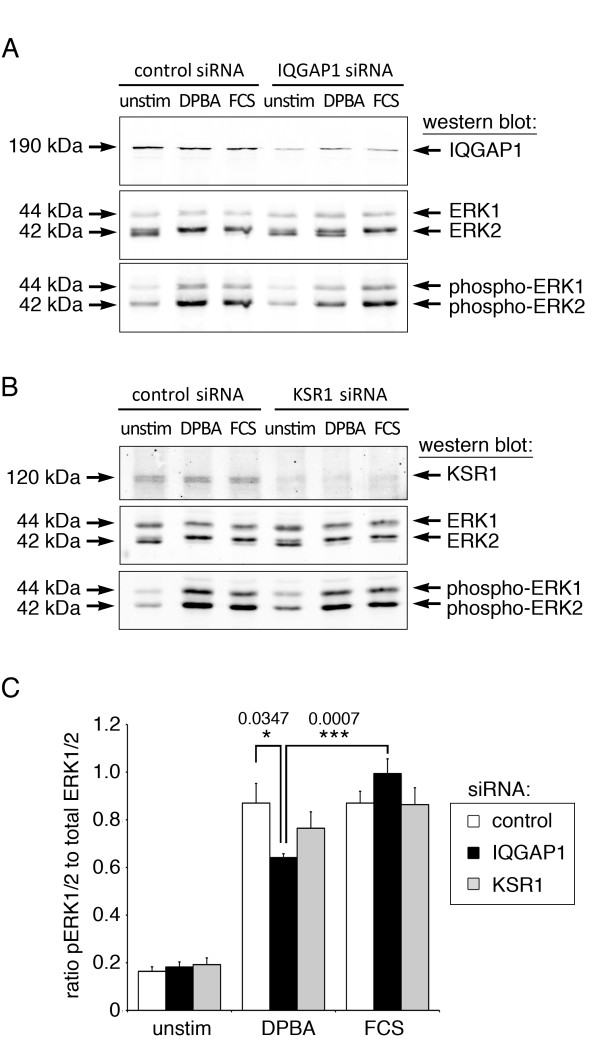
**ERK1/2 scaffold IQGAP1 mediates stimulus-specific ERK1/2 activation.** Cells transfected with siRNA directed against IQGAP1 or KSR1, or with undirected control siRNA, were stimulated with DPBA or FCS, or left unstimulated. **(A** and **B)** Western blot analysis of cell lysates from **(A)** IQGAP1 knock down and **(B)** KSR1 knock down experiments. **(C)** Densitometric analysis of ERK1/2 activation after siRNA knock down. Significance (*) and p-values are indicated on chart; error bars represent standard errors.

To test whether IQGAP1 and KSR1 mediate stimulus-specific activation of cytoskeletal ERK1/2, cells were transfected with siRNA directed against either IQGAP1 or KSR1. As a control, undirected siRNA was used (Dharmacon, Lafayette, CO). Four days after transfection, cells were either left unstimulated, or stimulated with DPBA or FCS. Whole cell lysates were analyzed for expression of IQGAP1, KSR1, ERK1/2, phospho-ERK1/2, and GAPDH. Figure 3 shows that DPBA-induced, but not serum-induced, ERK1/2 phosphorylation is significantly reduced after siRNA knock down of IQGAP1 (Figure 3A,C), pointing to a stimulus-specific function of this scaffold protein in ERK1/2 activation. Since ERK1/2 can be activated by several different pathways, interference with one pathway by knock down of one scaffold is not expected to abolish ERK1/2 activation completely. Hence, the small, but significant decrease of ERK1/2 activation by IQGAP1 after DPBA stimulation indicates that phorbol ester stimulation activates both IQGAP1-dependent and -independent of ERK1/2 pathways.

We found no effect of KSR1 knock down on phorbol ester or serum induced ERK1/2 phosphorylation (Figure 3B,C). This is in contrast to findings from other cell types and tissues, such as thymocytes, hippocampal tissue or an endometrial cancer cell line, where KSR1 has been shown to mediate phorbol ester- or serum-induced ERK1/2 activation [23-25]. The scaffolding activity or KSR1 and other scaffold proteins depends on the stoichiometry between the scaffold and its substrates [26]. Expression below or above the optimal stoichiometry can therefore result in inhibition instead of activation, as reported for overexpression of KSR1 in human embryonic kidney cells [27]. Nemoto et al. [28] have shown that in smooth muscle from diabetic aorta, ERK1/2 activation is elevated due to enhanced KSR1 activity compared to healthy aortic smooth muscle. This suggests that in healthy aorta cells, the scaffolding activity of KSR1 is submaximal, which could explain why in A7r5 cells, which are derived from healthy aortic smooth muscle, further reduction of KSR1 scaffolding activity by siRNA knock down does not result in a noticeable effect on ERK1/2 activation.

### IQGAP1 mediates actin association of ERK1/2

**Figure 4:**
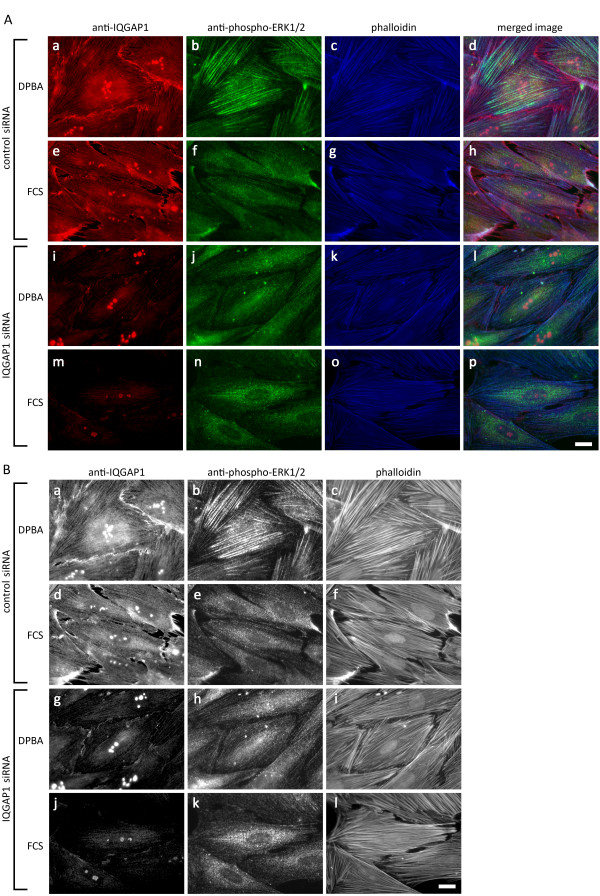
**IQGAP1 is required for activation of cytoskeletal ERK1/2. (A)** IQGAP1 knock down interferes with filamentous localization of phospho-ERK1/2. A7r5 cells on coverslips transfected with siRNA directed against IQGAP1 were stimulated with DPBA or FCS. After fixing, cells were co-stained for IQGAP1 (red channel) and phospho-ERK1/2 (green channel); filamentous actin was stained with Alexa350-phalloidin (blue channel). The panels show representative images from one out of three independent experiments. **(B)** The same images as in (A) shown in black and white for enhanced contrast. Scale bar, 20 μm.

As an actin binding protein [29], IQGAP1 is an obvious candidate for tethering ERK1/2 to actin. To test this idea, we imaged phospho-ERK1/2 by immunofluorescence microscopy after siRNA knock down of IQGAP1. Non-targeting siRNA was used as control. Pre-fixing Triton X-extraction was used to remove soluble ERK1/2. As shown in Figure 4, IQGAP1 expression was markedly reduced after transfection with siRNA directed against IQGAP1 (Figure 4A, h and l and B, g and j), but not in the control siRNA transfected cells (Figure 4A, a and e, B, a and d). In the control siRNA transfected cells, filamentous association of phospho-ERK1/2 was observed after DPBA stimulation (Figure 4A, b, and B, b), but not after serum stimulation (Figure 4A, f and B, e). After siRNA knock down of IQGAP1, however, no filamentous staining of ERK1/2 was seen after serum or phorbol ester stimulation (Figure 4A, j and n, and B, h and k).

**Figure 5:**
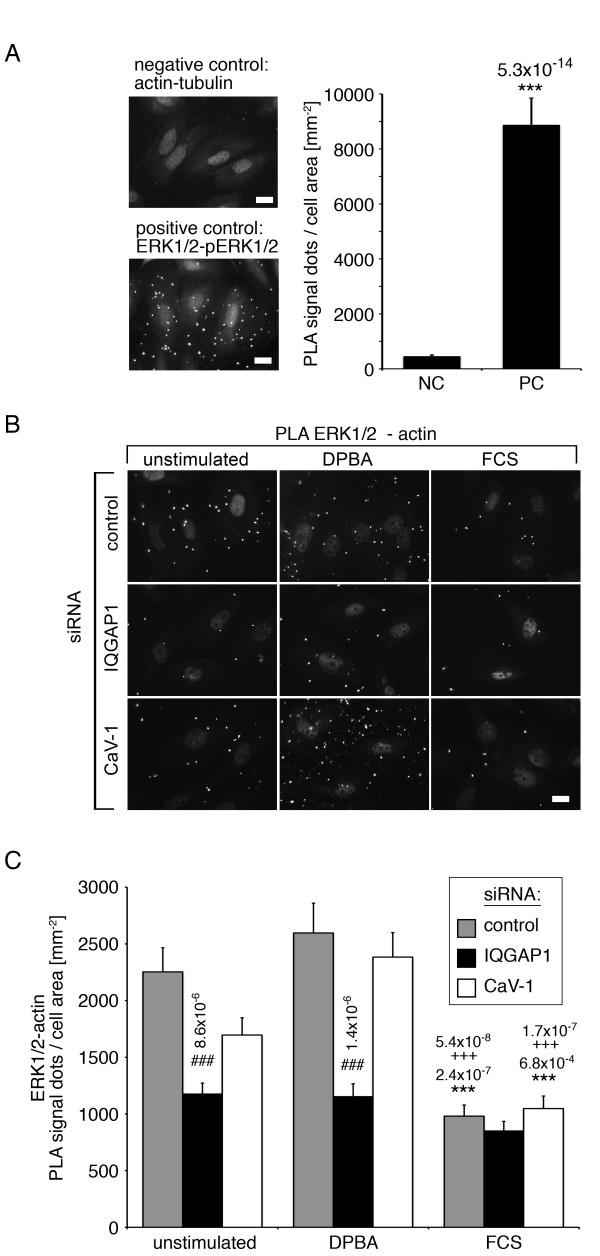
**IQGAP1 mediates actin association of ERK1/2.** Cells were transfected with siRNA as indicated. Before fixing, cells were stimulated with either FCS or DPBA, or left unstimulated. Proximity between ERK1/2 and actin was analyzed by proximity ligation assays (PLA). **(A)** Immunofluorescence and statistical analysis of PLA with a negative control (anti-actin and anti-tubulin) and positive control (anti-ERK1/2 and anti-phospho-ERK1/2). **(B)** Immunofluorescence images of the ERK1/2-actin PLA pair show that IQGAP1 siRNA reduces actin association of ERK1/2. **(C)** Statistical analysis of 60 cells for each test from three independent experiments (*compared to unstimulated samples; # compared to corresponding control samples; + between DPBA and FCS; error bars represent standard errors)**.** Scale bar, 20 μm.

To quantify these results, we employed in situ proximity ligation assays (PLA) in siRNA-transfected cells. PLA produces fluorescent dots if pairs of proteins are within 30 nm of each other [30]. The signal range was determined in control experiments using unstimulated cells tested for actin-tubulin proximity as negative control, and serum stimulated cells tested for ERK1/2-phospho-ERK1/2 proximity as positive control (Figure 5A).

Figure 5B and C show immunofluorescence images with PLA signal dots for ERK1/2 and actin PLA pairs, and the statistical analysis of these data. Since we have previously shown that similar to IQGAP1 siRNA, caveolin-1 siRNA also caused reduced filamentous phospho-ERK1/2 staining in immunofluorescence experiments [11], we included caveolin-1 siRNA in the PLA analysis. In cells transfected with undirected control siRNA or caveolin-1 siRNA, both unstimulated and DPBA stimulated cells show PLA signals well above the background signal (compare negative control in Figure 5A), indicating close proximity of ERK1/2 and actin in these cells, whereas the PLA signal is significantly lower after FCS stimulation, which is in line with the previously shown dissociation of ERK1/2 from the actin filaments upon serum stimulation [11]. Importantly, transfection with IQGAP1 siRNA leads to significantly reduced PLA signals in unstimulated and DPBA stimulated cells, showing that IQGAP1 is required for cytoskeletal association of ERK1/2 in unstimulated and in phorbol ester stimulated cells. The displacement of ERK1/2 from filamentous actin after serum stimulation, as indicated by the reduced PLA signal for the actin-ERK antibody pair after serum stimulation, is in agreement with our findings that after serum stimulation, the interaction between IQGAP1 and ERK1/2 is reduced compared to unstimulated cells (Figure 2).

We have shown previously shown that a Triton X-insoluble fraction of ERK1/2 moves into the Triton X-soluble fraction after serum stimulation, but not after phorbol ester stimulation [11]. These findings, taken together with the data presented here, suggest that binding of ERK1/2 to IQGAP1 restricts ERK1/2 solubiliy and hence, shifts ERK1/2 signaling to extranuclear, non-proliferative functions [31,32].

### Caveolin-1 and IQGAP1 are upstream and downstream scaffolds of the same ERK1/2 activation pathway

**Figure 6:**
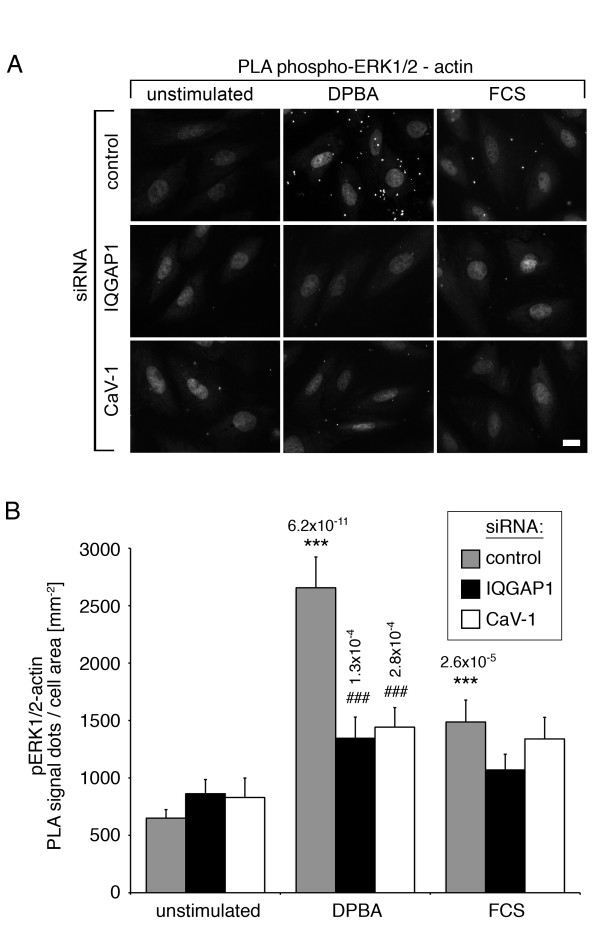
**IQGAP1 and caveolin-1 are required for activation of cytoskeletal ERK1/2.** Cells were transfected with siRNA as indicated. Before fixing, cells were stimulated with either FCS or DPBA, or left unstimulated. Proximity between phospho-ERK1/2 and actin was analyzed by proximity ligation assays (PLA). **(A)** Immunofluorescence images of the phospho-ERK1/2-actin pair show that both, caveolin-1 and IQGAP1, are required for phosphorylation of actin-associated ERK1/2. **(B)** Statistical analysis of 60 cells for each test from three independent experiments (*compared to unstimulated samples; # compared to corresponding control samples; + between DPBA and FCS; error bars represent standard errors)**.** Scale bar, 20 μm.

**Figure 7:**
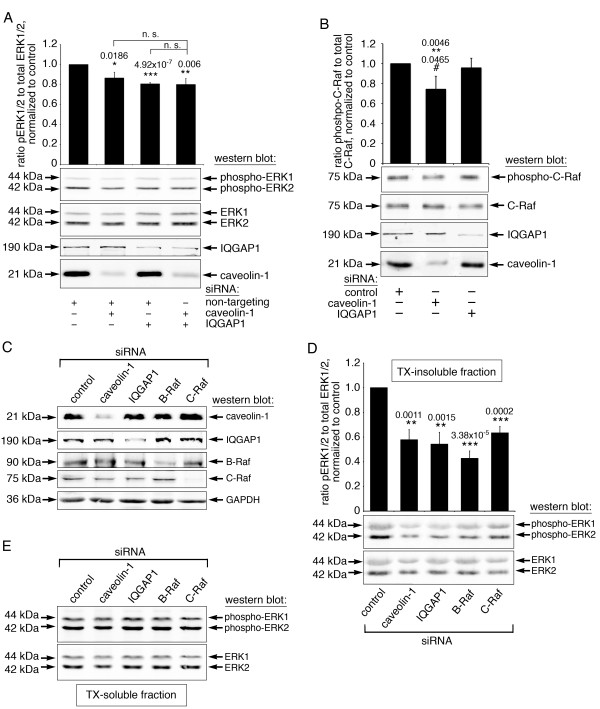
**IQGAP1 and caveolin-1 are upstream and downstream scaffolds in the same ERK1/2 activation pathway. (A)** Double knock down of IQGAP1 and caveolin-1 shows the same decrease in DPBA-induced ERK1/2 activation as single knock down. Cells were transfected with siRNA as indicated, then stimulated with DPBA for 5 minutes. Lysates were subjected to western blotting for analysis of ERK1/2 phosphorylation. **(B)** C-Raf activation is reduced after knock down of caveolin-1, but not after knock down of IQGAP1. Cells were transfected as indicated, then stimulated with DPBA for 5 minutes. The ratio of phospho-C-Raf (S338) to total C-Raf was analyzed on duplicate membranes after normalization to GAPDH. **(C-E)** To enrich the cytoskeletal fraction, DPBA stimulated A7r5 cells were subjected to Triton X-extraction before preparation of soluble and insoluble cell extracts. Insoluble and soluble lysates were analyzed for ERK1/2 phosphorylation as well as target protein expression by western blotting and densitometry. Data represent five independent experiments. **(C)** Western blots show siRNA knock down of caveolin-1, IQGAP1, B-Raf and C-Raf. Insoluble samples are shown for caveolin-1 and IQGAP1 expression, soluble samples are shown for B-Raf, C-Raf and GAPDH. **(D)** The graph shows average ERK1/2 phosphorylation in the TX-insoluble fraction, along with a representative western blot. **(E)** The western blot shows phosphorylated ERK1/2 and total ERK1/2 in TX-soluble samples. Significance compared to control (*) and compared to IQGAP1 siRNA (#), as well as p values are indicated on graphs; error bars represent standard errors.

An analogous set of PLA experiments was performed to test for proximity between phosphorylated ERK1/2 and actin. As shown in Figure 6A, with quantitative analysis in Figure 6B, the PLA signal is strongly enhanced in control cells after DPBA stimulation compared to unstimulated or FCS stimulated cells, showing that actin associated ERK1/2 is specifically activated in response to phorbol ester stimulation. The small, but significant increase in phospho-ERK1/2-actin proximity events after FCS stimulation compared to unstimulated cells might be caused by co-activation of PKC. Of note, activation of actin associated ERK1/2 is prevented by transfection with either IQGAP1 siRNA or caveolin-1 siRNA. The PLA data suggest that IQGAP1 and caveolin-1 both act as ERK1/2 scaffolds in the same phorbol ester induced ERK1/2 activation pathway. To further test this idea, we transfected cells with various siRNA combinations and analyzed ERK1/2 activation after phorbol ester stimulation. As shown in Figure 7A, total cellular ERK1/2 phosphorylation is significantly reduced after knock down of caveolin-1, IQGAP1 or a combination of both. Notably, ERK1/2 phosphorylation after simultaneous knockdown of caveolin-1 and IQGAP1 is not significantly different from ERK1/2 phosphorylation after knockdown of either caveolin-1 or IQGAP1 alone. We conclude from the non-additive effect of scaffold knockdown on ERK1/2 activation, that caveolin-1 and IQGAP1 act in the same pathway.

In the PLA experiments, both IQGAP1 and caveolin-1 are required for ERK1/2 activation in response to DPBA. That IQGAP1 was also required for actin association, suggests that IQGAP1 interacts with ERK1/2 and actin directly and simultaneously, which eliminates the need for any further downstream scaffold protein. Since caveolin-1 knock down, like IQGAP1 knock down, also reduced ERK1/2 activation in response to DPBA, it is therefore most likely positioned upstream of IQGAP1 in this pathway. To further elucidate the hierarchical arrangement of caveolin-1 and IQGAP1, we assayed for activation of Raf. Since C-Raf associates with caveolin-1 [9] and is known to mediate phorbol ester induced ERK1/2 activation in various cell types [33-35], we analyzed C-Raf activation in response to DPBA after transfection with control siRNA or siRNA directed against either caveolin-1 or IQGAP1. We used phosphorylation at serine 338 as an indicator of C-Raf activation [36]. The results presented in Figure 7B show that only caveolin-1 knock down significantly reduces C-Raf phosphorylation, confirming that caveolin-1, but not IQGAP1, is upstream of C-Raf in this signaling pathway.

Knock down of caveolin-1, IQGAP1, B-Raf and C-Raf reduces activation of cytoskeletal ERK1/2, but not soluble ERK1/2, after DPBA stimulation

To find out to what extent caveolin-1 and IQGAP1 scaffolding activites as well as B-Raf and C-Raf signaling are required for activation of cytoskeletal ERK1/2, we enriched actin-associated ERK1/2 by subcellular fractionation. In these experiments, cells were transfected with siRNA directed against caveolin-1, IQGAP1, B-Raf and C-Raf. Non-targeting control siRNA (Dharmacon) was used as control. Four days after transfection, cells were stimulated with DPBA and then subjected to Triton X-100 (TX) extraction before preparation of soluble and insoluble lysates. Target protein knock down was confirmed by western blots (Figure 7C). ERK1/2 phosphorylation was analyzed in the TX-insoluble and soluble fractions (Figure 7D and E). Densitometric analysis revealed that in the insoluble fraction, siRNA knock down of caveolin-1, IQGAP1, B-Raf and C-Raf decreased ERK1/2 phosphorylation significantly to 45-60% of the control samples (graph in Figure 7D). Interestingly, no significant decrease of ERK1/2 phosphorylation was found for any of the tested siRNAs in the soluble fraction (data not shown). This result shows that the effect of the different siRNAs is restricted to the insoluble cytoskeletal fraction, which further substantiates that caveolin-1, IQGAP1, B-Raf and C-Raf all participate in the same pathway that lead to activation of actin-associated ERK1/2.

### B-Raf and C-Raf heterodimerization as possible link between caveolin and IQGAP1

**Figure 8:**
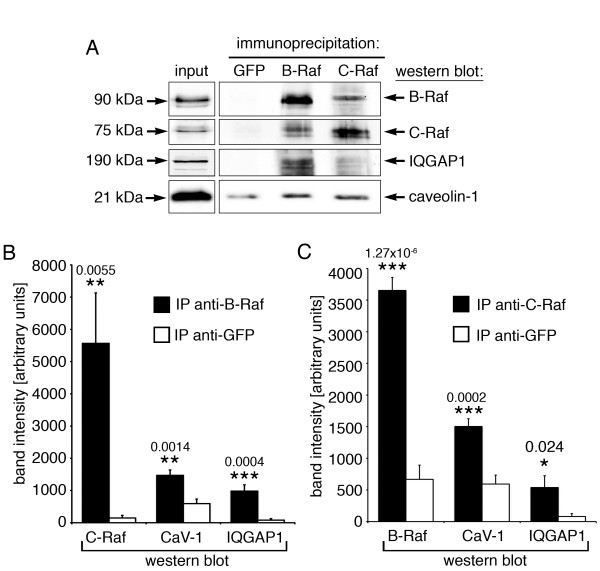
**B-Raf and C-Raf interactions in A7r5 smooth muscle cells. (A)** Interactions between Raf isoforms, IQGAP1 and caveolin-1 were assessed by immunoprecipitation experiments. Lysates were immunoprecipitated with an anti-GFP antibody as control, or with an anti-B-Raf or anti-C-Raf antibody. Co-immunoprecipitated B-Raf, C-Raf, caveolin-1 and IQGAP1 were detected by western blotting. **(B)** Statistical analysis of co-immunoprecipitated C-Raf, caveolin-1 and IQGAP1 from 7 independent IPs with the anti-B-Raf antibody (GFP shown as control). **(C)** Statistical analysis of co-immunoprecipitated B-Raf, caveolin-1 and IQGAP1 from 7 independent IPs with the anti-C-Raf antibody (GFP shown as control). Significance compared to control (*) and p values are indicated on graphs; error bars represent standard errors. n. s., not significant.

IQGAP1 has been demonstrated to bind to B-Raf [37], whereas caveolin-1 has been reported to bind to C-Raf [9,38]. We are not aware of reports demonstrating cellular endogenous protein-protein interactions between caveolin-1 and B-Raf, or IQGAP1 and C-Raf, however, recombinant IQGAP1 fragments have been shown to bind to C-Raf [39]. Hence the question arises as to how caveolin-1 and IQGAP1 are linked. Three possibilities come immediately to mind: interaction between caveolin-1 and B-Raf, IQGAP1 and C-Raf, or B-Raf and C-Raf. To test these possibilities, we performed co-immunoprecipitation experiments with anti-B-Raf and anti-C-Raf antibodies, along with an anti-GFP-antibody as control. As shown in Figure 8A-C, we detected a robust co-immunoprecipitation of C-Raf in anti-B-Raf immunoprecipitations (Figure 8B and C), along with smaller amounts of co-precipitated caveolin-1 and IQGAP1. Similarly, in C-Raf immunoprecipitations (Figure 8A and C), we found co-precipitated B-Raf along with smaller amounts of caveolin-1 and IQGAP1. Since interaction between the two Raf isoforms appeared to be more robust than interaction of either Raf isoform with caveolin-1 or IQGAP1, and based on our finding that both B-Raf and C-Raf are required for DPBA induced activation of cytoskeletal ERK1/2 (Figure 8D), we speculate that the signaling modules scaffolded by caveolin-1 and IQGAP1 are most likely linked via Raf heterodimerization, and that some of the weaker interactions could represent indirect interactions.

### Suggested model for hierarchical scaffolding by caveolin-1 and IQGAP1

**Figure 9:**
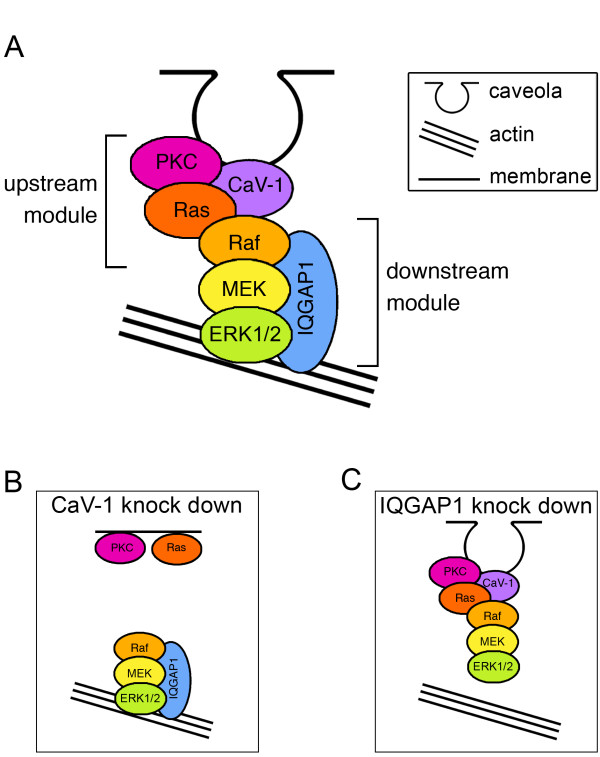
**Model for hierarchical scaffolding by caveolin-1 and IQGAP1. (A)** We suggest a model in which an upstream signaling module, associated with caveolin-1 and consisting of PKC, Ras, and C-Raf, is linked via Raf heterodimerization to a downstream signaling module, scaffolded by IQGAP1 and consisting of B-Raf, MEK and ERK. **(B)** Knock down of caveolin-1 prevents activation of actin-associated ERK1/2 by PKC, but does not interfere with actin association of ERK1/2. **(C)** Knock down of IQGAP1 disconnects ERK1/2 from actin.

Based on our results, we present a model in which caveolin-1 and IQGAP1 act as scaffolds in the same PKC-induced ERK1/2 activation pathway, with caveolin-1 facilitating signaling along the upstream part of the pathway, and IQGAP1 tethering the downstream part of the signaling cascade to the actin cytoskeleton (Figure 9A). After caveolin-1 knock down (Figure 9B), cytoskeletal ERK1/2 would still be in place, but it would not be activated in response to activation of PKC. In contrast, after IQGAP1 knock down (Figure 9C), ERK1/2 could still be activated in a complex with caveolin-1, but it would not be associated with the actin filaments.

We do not know yet the precise spatiotemporal arrangement of this signaling complex. The possibilities include (1) direct interaction of caveolin-1 and IQGAP1, (2) sequential or (3) simultaneous interaction with the Raf-MEK-ERK cassette, possibly through dimerization of Raf, MEK and/or ERK [40]. Of note, IQGAP1 has been implicated in stabilizing caveolae [41], indicating a functional interaction. However, we were not able to detect a physical interaction between IQGAP1 and caveolin-1 in immunoprecipitation experiments, rendering option (1) the least likely of the three. The previously observed dissociation of activated ERK1/2 from caveolae [11] argues for option (2), as it could be interpreted as handing over of activated ERK1/2 from the caveolae to the actin cytoskeleton. Ren et al. [37] have placed IQGAP1 upstream of B-Raf, whereas we found IQGAP1 downstream of C-Raf. This apparent contrast could be explained by option (3), assuming that C-Raf, which is activated via caveolin-1, heterodimerizes with B-Raf, which is associated with IQGAP1. Indeed we demonstrate robust B-Raf and C-Raf heterodimerization in endogenous immunoprecipitation experiments (Figure 8). Thus, Raf heterodimerization could be here, and possibly in other pathways, a contact point between upstream and downstream signaling modules. Indeed, Raf heterodimerization is emerging as an important factor for Raf signaling activity [42-46]. Moreover, it has been shown that Raf heterodimerization yields an even more active complex than a Raf homodimer, and that only one Raf molecule in the dimer needs to be active [46]. In the scenario of option (3), negative feedback regulation of activated ERK1/2 on B-Raf [47] could therefore be overridden by positive feedback via Cdc42 and p21 activated kinase, which activates C-Raf [36], as long as the complex is bound to actin. Further experiments are needed to investigate these multiple detailed possibilities.

In the few currently known examples of functional interaction between scaffolds, scaffolds cooperate in a complementary manner, rather than in a hierarchical arrangement as shown here. In the drosophila Hippo pathway for example, activation of the transcription factor Warts (Wts) requires the concerted action of the scaffold proteins Sav and Mats; as another example, the scaffold proteins BNIP-2 and JLP are required simultaneously for activation of p38 by cdc42 in myogenic and neuronal differentiation [1].

## Conclusions

Scaffold proteins group signaling proteins into signaling modules. Here, we show how, on a higher level, two scaffold proteins functionally interact to link two signaling modules. Our data show that the phorbol ester stimulated pathway PKC-Ras-Raf-MEK-ERK is broken down into two modules, of which one, PKC-Ras-(C-)Raf, is assembled at the caveolae with the help of caveolin-1, and the other one, (B-)Raf-MEK-ERK, is tethered to the actin cytoskeleton with the help of IQGAP1. Enhanced proliferative activation of ERK signaling is held responsible for the oncogenic effect of Ras or B-Raf mutations, which are found at a high incidence in many types of human cancers [48-50]. As signaling regulators, scaffold proteins could be useful in developing therapeutic approaches to interfere with unwanted ERK signaling (i.e., proliferative pathways), while at the same time preserving other ERK pathways (e.g. differentiation, apoptosis, contraction). For this purpose, the contact points between signaling modules could be uniquely suited targets for drug discovery programs.

## Methods

### Reagents and antibodies

General laboratory reagents were of analytical grade or better and were purchased from Sigma (St. Louis, MO) and Bio-Rad (Hercules, CA). For stimulation, fetal calf serum (FCS, Invitrogen, Carlsbad, CA) was used at 10% and 12-deoxyphorbol 13-isobutylate 20-acetate (DPBA, LC Laboratories, Woburn, MA) was used at 3 μmol/L. Duration of stimulation was 5 minutes in all experiments. Since DPBA was dissolved in dimethylsulfoxide (DMSO), which may affect ERK1/2 activation [51-53], unstimulated cells and serum stimulated cells were treated with the corresponding amount of DMSO (0.03%). The following primary antibodies were used for western blots: mouse monoclonal anti-ERK1/2 (1:500, Cell Signaling, Danvers, MA), rabbit polyclonal anti-phospho-ERK1/2 (1:2000, Cell Signaling), mouse monoclonal anti-KSR1 (1:100, BD Biosciences, San Diego, CA), rabbit polyclonal anti-IQGAP1 (1:500, Santa Cruz Biotechnology, Santa Cruz, CA), rabbit polyclonal anti-C-Raf antibody (1:250, Cell Signaling), rabbit monoclonal anti-phospho-C-Raf (1:250, serine 338; Cell Signaling), rabbit polyclonal anti-B-Raf antibody (1:500, Santa Cruz), rabbit anti-caldesmon antibody (1:500, Abgent), anti-beta1-integrin-antibody (1:500, Cell Signaling) and rabbit polyclonal anti-GAPDH antibody (1:200,000, Sigma). For immunofluorescence microscopy and proximity ligation assays, following ERK1/2 antibodies were used: mouse monoclonal anti-phospho-ERK1/2 (1:200, Cell Signaling), rabbit polyclonal anti-ERK1/2 (1:200, Cell Signaling). For all other proteins, the same antibodies as listed for western blot analysis were used. As secondary antibodies in immunofluorescence experiments, goat anti-rabbit and goat anti-mouse Alexa Fluor® 488 and Alexa Fluor® 568 (1:1000, Invitrogen) were used. IRDye® 680 or IRDye® 800CW labeled goat anti-rabbit or goat-anti-mouse IgGs were used as secondary antibodies in western blot experiments (1:1000, LI-COR, Lincoln, NE).

### Cell culture and siRNA transfection

A7r5 rat aorta cells (ATCC, Manassas, VA) were cultured in DMEM high glucose (Invitrogen) with 10% FCS, 1% glutamine, 50 units/ml penicillin and 50 μg/ml streptomycin. Cells were grown to confluency and incubated in medium containing 0% serum for 24 h prior to all experiments to ensure differentiation of the cells to the smooth muscle-like phenotype [54,55]. SiRNA oligonucleotides for knock down of rat caveolin-1 have been described (Vetterkind 2012); siGenome smartpool siRNA was used for knock down of rat IQGAP1, rat KSR1, rat B-Raf and rat C-Raf (Dharmacon, Lafayette, CO). A mix of four nontargeting siRNAs (non-targeting siRNA pool #2, Dharmacon) was used as control. Transfection with 40 nmol/L siRNA molecules was performed with Lipofectamine 2000 (Invitrogen) according to the manufacturer’s instructions. Cells were processed for experiments 5 days after siRNA transfection.

### Immunofluorescence imaging and proximity ligation assay (PLA)

Cells were fixed and stained as previously described [56]. For imaging of cytoskeletal phospho-ERK1/2, cells were pre-extracted for 3 minutes at 37°C with 0.25% Triton X-100 in PIPES/EGTA/MgCl_2_ (PEM) buffer (80 mM PIPES, pH 6.8, 1 mM EGTA, 1 mM MgCl_2_, 4% PEG). Cells were examined with an Eclipse TE2000-E fluorescence microscope (Nikon, Melville, NY) equipped with a CCD camera and using filters optimized for double-label experiments. Images were optimized for display with Photoshop CS3 software (Adobe Systems, Mountain View, CA). For proximity ligation assays (PLA), fixed cells were stained with primary antibodies as indicated and subsequently stained essentially according to the manufacturer’s instructions [57]. For each antibody pair, 60 cells from (20 each from three independent experiments) were analyzed. Analysis was performed using NIS Elements AR 2.30 software (Nikon, Melville, NY). The lower threshold for dot detection was set to three times background level. Further, dot detection was limited by size (maximum 0.5 μm) and circularity restrictions (minimum 0.5).

### Cell extracts

Prior to preparation of cell lysates, cells were either stimulated with DPBA (3 mmol/L) or with FCS (10%) for five minutes, or left unstimulated. To prepare whole cell extracts, plates were washed with ice-cold phosphate buffered saline (pH 7.2) and then scraped off in lysis buffer (mmol/L: 140 NaCl, 3 MgCl_2_, 1 dithiothreitol and 0.5% Nonidet-P40 in a 20 mmol/L sodium phosphate buffer, pH 8.0) or IP lysis buffer (50 mmol/L NaCl, 10% glycerol, 1% Nonidet-P40 in a 10 mmol/L sodium phosphate buffer, pH 8.0) supplemented with protease inhibitor cocktail (Roche, Indianapolis, IN). Cells were lysed on ice for 30 min. Lysates were cleared by centrifugation (16,000 rcf, 10 minutes at 4°C).

### Immunoprecipitation and western blot

For immunoprecipitation experiments, A7r5 lysates (in IP lysis buffer) were incubated with anti-IQGAP1, anti-KSR1, anti-B-Raf or anti-C-Raf primary antibodies (see “Reagents and antibodies”) cross-linked to Protein G-dynabeads® (Invitrogen) or with anti-ERK1/2 cross-linked to protein A agarose beads (Millipore) at 4°C over night. The immobilized antigen-antibody complexes were washed three times with IP lysis buffer and eluted in sample buffer. Proteins in the samples were separated on 12.5% SDS polyacrylamide gels according to standard procedures. For western blot analysis, proteins on SDS gels were transferred onto nitrocellulose membranes (Whatman, Florham Park, NJ). Bound proteins were detected with specific primary antibodies and appropriate secondary antibodies. Bands were visualized on an Odyssey® infrared imaging system (LI-COR). Densitometry analysis was perfomed on raw data with the Odyssey 2.1 software. For analysis of protein expression, bands of interest were normalized to GAPDH on the same membrane. For analysis of protein phosphorylation, phospho-ERK1/2 and total ERK1/2 were analyzed in parallel on the same membrane, and phospho-C-Raf and total C-Raf were analyzed on duplicate membranes after normalization to GAPDH. For statistical analysis of immunoprecipitation experiments, background signal as detected in control immunoprecipitations was either subtracted from immunoprecipitated protein bands (Figure 2A-D) or control IPs are shown along with the experimental IPs (Figure 7C-E). Co-immunoprecipitated protein band intensities were then normalized to immunoprecipitated target protein. For the experiments shown in Figure 2, band intensities were further normalized to unstimulated samples. Ponceau staining was used to monitor equal protein loading and transfer.

### Subcellular fractionation

Subcellular fractionation for differential ultracentrifugation was performed as described previously [58]. Briefly, cells were homogenized by 5 gentle strokes with a 22-gauge hamilton syringe in buffer A (20 mmol/L Tris–HCl, pH 7.5, 250 mmol/L sucrose, 10 mmol/L dithiothreitol, 3 EGTA mmol/L, 5 mmol/L MgCl_2_, 1 mmol/L ATP) supplemented with 50 mmol/L NaCl. Cell homogenates were centrifuged at 100,000 g for 1 hour and the supernatant collected as the cytosolic fraction. The pellet was resuspended in buffer A supplemented with 0.5% Triton X-100, extracted at 4°C for 1 hour and centrifuged at 100,000 g for 1 hour. This supernatant was collected as the Triton X-soluble or membrane fraction. The pellet was resuspended in buffer A with 0.5% Triton X-100 and 1.2% SDS, extracted at 4°C for 1 hour and briefly centrifuged. This final supernatant was collected as the Triton X-insoluble or cytoskeletal fraction. Equal volumes of buffers I-III were used. All buffers were supplemented with protease inhibitor cocktail (Roche, Indianapolis, IN) and homogenates were kept on ice or at 4°C between centrifugations to prevent proteolysis. Triton X soluble and -insoluble fractions were prepared as described previously [11]. Briefly, plates were washed once with prewarmed (37C) PEM buffer (80 mM PIPES, pH 6.8, 1 mM EGTA, 1 mM MgCl_2_, 4% PEG). Cells were scraped off in prewarmed PEM buffer with 0.25% Triton X-100 and incubated at 37**°**C for 3 minutes with gentle agitation. After centrifugation (400 rcf at room temperature) for 2 minutes, the supernatant was collected and the pellet was resuspended in sample buffer (= Triton X-100 insoluble fraction). Proteins in the supernatant (=Triton-X soluble fraction) were precipitated over night after adding 2.5 volumes of ethanol. Precipitated protein was pelleted by centrifugation (16,000 rcf, 10 minutes at 4**°**C) and resuspended in sample buffer.

### Statistical analysis

All values given in the text and displayed in the graphs are mean ± standard error. Differences between means were evaluated using two-tailed Student’s t-tests. Western blots were analyzed by densitometry using an Odyssey infrared scanner (LiCor). Data from at least four independent experiments were used for statistical analyses. For proximity ligation assay (PLA) experiments, because of the high number of analyzed cells (n=60) significance was taken at the p<0.001 level to minimize type I errors. In all other analyses, differences were considered significant at the p<0.05 level.

## Abbreviations

DPBA: 12-deoxyphorbol 13-isobutylate 20-acetate; ERK1/2: Extracellular signal regulated kinase 1 and 2; FCS: Fetal calf serum; IQGAP1: IQ domain containing GTPase activating protein 1; KSR1: Kinase suppressor of ras 1; MAPK: Mitogen activated protein kinase; PKC: Protein kinase C.

## Competing interests

The authors declare that they have no competing interests.

## Authors’ contributions

SV and KGM designed the project, analyzed the data and wrote the manuscript. SV designed the experiments. SV, RP and QQL performed the experiments. All authors read and approved the final manuscript.
